# Dr. Thomson's New Theory of Digestion

**Published:** 1829-07-01

**Authors:** 


### XLVI.

NEW THEORY OF DIGESTION. By Dr. THOMSON, Professor of Chemistry in the University of Glasgow.

This paper, which is published in our respected Glasgow cotemporary of the present quarter, has afforded us considerable amusement. It is a fine illustration of the bias produced in men's minds by the nature of their studies or their avocations. Thus, the anatomist seeks for the *solution* of a problem (in this instance the *solution* of roast beef or plumb pudding) in animal organization or animal secretion—as, for example, the glands of the stomach and the gastric juice. The experimental physiologist solves the difficulty by means of nervous influence, vital power, the electric fluid, or some other subtile agent:—While the chemical philosopher disengages some acid or alkali that effects the object—say digestion—in the twinkling of an eye.

It is necessary that the constructor of a new theory should, as a matter of course, demolish all those which have preceded him. And this, fortunately, is so easy, that it seldom requires more than a few minutes for the operation. A theory is as readily strangled as a man—and though it cannot be always done without pain to some of the parties concerned, it is unaccompanied by any legal penalty, save that of an occasional literary warfare.

Dr. Thomson is too systematic a philosopher to depart from established custom; and, therefore, he demolishes all that stands in his way before he erects his own edifice—just in the same way as the old houses in the Strand are dilapidated preparatory to the formation of the new line, which seems to spring up, as if by enchantment, in their rear.*

Dr. Thomson treats *fermentation and trituration* with lenient, but sovereign contempt, and only condescends to open his battery for a few minutes on the *gastric juice*, as the "presently received explanation" of digestion. As we are fond of jumping at conclusions, we shall not trouble our readers with the arguments which Dr. T. adduces against the existence of a solvent gastric juice. To us, indeed, these arguments do not appear very strong; but still we are more disposed to give an account of the constructive than the destructive theory.

The experiments of Stevens, Reaumur, and Spalanzani, shewing the solution of substances inclosed in metal tubes, and introduced into the stomach, might appear rather hard of digestion for the new theory, which denies the existence of a *solvent* juice in the stomach; but no:—every thing is "easy to the ethereal mind."

"Now, as food could not have been dissolved without a solvent, it was concluded that a solvent was secreted by the stomach itself, and this solvent, therefore, was called the *gastric juice*."

Our author admits that the experiments of the physiologists above-mentioned, furnish irresistible proof of the existence of a gastric juice; but Dr. T. is of opinion "that the *solvent* is not secreted by the stomach, under the form of gastric juice; but that it exists in the

---

* How often have we lingered to survey the modern Golgotha (or place of sculls) in the Strand, contemplating the geography of our faculties on the intellectual maps in Mr. Deville's window! All at once we missed our favourite lounge, and found nothing but mouldering ruins and carts in its stead. Two or three days afterwards, we were delighted to see a new and splendid Golgotha in the rear, as if erected by Aladdin's lamp—or the lamps of Deville, and where we hope soon to see the head of our worthy friend, Mr. Nash, with the organs of *constructiveness* and *destructiveness* fully developed. By the way, we anticipate some difficulty in the doctrines of phrenology, in consequence of the new improvements. Looking at the Metropolis during the last ten or fifteen years, it would be difficult to determine which organ predominates in the heads of our great architects. For ourselves, we have no doubt that "DESTRUCTIVENESS" has the priority—CONSTRUCTIVENESS the superiority.

food, and is merely isolated by the stomach, and brought into a state capable of acting as a solvent." The following are the facts upon which this opinion is founded.

"It will probably be admitted that common salt is indispensible as a seasoner of food. It has been employed in all ages, and in every country, for that purpose; or if there be any nation which does not employ it, we shall find that this nation lives at no great distance from the sea, and that the food and water used by them, contain enough of salt to answer all the purposes which nature has in view. For common salt is an almost constant ingredient in spring and river water, and all the animal and vegetable substances used for the food of man contain either common salt, or an alkaline chloride of some kind or other. This universal use of common salt cannot be accounted for, unless we admit it to be essential to the proper digestion of food.

"Vauquelin announced, many years ago, that the stomachs of oxen, sheep, and calves always contain a quantity of uncombined acid, which he considered as the phosphoric. But Dr. Prout has shown, by decisive experiments, that this acid is the *muriatic*, and that the human stomach in all cases contains a quantity of uncombined muriatic acid. He found the same uncombined acid constantly in the stomach of the hare, horse, calf, and dog. These observations have been amply confirmed by Tiedeman and L. Gmelin, who examined the liquid in the stomachs of no fewer than 43 animals, dogs, cats, horses, oxen, calves, and sheep. The liquid in the stomach was acid in every case, and the quantity of acid was always considerable. Muriatic acid was always present. They found likewise *acetic acid*, and they say also *butyric acid*. These two last probably appear only in cases of imperfect digestion. For Dr. Prout found acetic acid as well as muriatic acid, in the liquid thrown up by dyspeptic patients.

"Tiedeman and Gmelin found, by experiment, that when food is put into the acid liquid from the stomach, and kept at the temperature of the human body, it is actually dissolved, and converted into chyme, just as it is in the living stomach. They also found that food, when exposed to the action of dilute solutions of muriatic acid, acetic acid, and acetate of soda, at the usual temperature of the living stomach, is speedily dissolved, and brought into a state resembling chyme.

"The stomach is supplied with nerves from the par vagum and the great sympathetic; and it is well known that the gastric nerves are large and numerous. Many experiments have been made on the effect produced on the stomach by cutting the par vagum. The uniform result has been, that the process of digestion was quite destroyed, the food remained in the stomach unaltered, and the animal, though supplied with food *ad libitum*, died from inanition. It may be sufficient to state the result of the trials of Dr. Wilson Philip in the hospital of Worcester. He found that if the par vagum be cut, and the cut extremities be allowed to remain *in situ*, the process of digestion is at first impeded; but it is slowly restored again; because in process of time, the cut extremities of the nerves again unite, and the stomach becomes capable of performing its function. But when the par vagum is cut, and a portion of it removed, so as to prevent the extremities from again coming in contact and uniting, the process of digestion ceases, and the function of the stomach is destroyed.

"Dr. Wilson Philip, after cutting out a part of the par vagum of a rabbit, charged a small galvanic battery, and by means of wires from each extremity, taking the stomach of the rabbit in their way, completed the chain of communication. By this contrivance, a current of electricity was passing constantly through the stomach. The result was, that the process of digestion was restored, and went on as well as if the par vagum had not been cut and removed. This important experiment was afterwards repeated successfully in London. Thus it has been ascertained, that electricity may be substituted for the nervous energy, in the process of digestion.

"*When a current of electricity, produced in a galvanic battery, passes through a liquid having any salt in solution, the salt is decomposed, the acid accumulating round the positive, and the alkaline base round the negative pole. There is reason to believe that it acts in this way in the stomach, when it is made to supply the place of the nervous energy. It would seem from this, that the nerves of the stomach act upon the liquids contained in the stomach, precisely as a galvanic current does; and probably by supplying a constant electrical current. By the nerves of the stomach, the common salt held in solution by the liquids in the stomach, is decomposed, and its muriatic acid set at liberty. The acid thus disengaged, acts upon the food and converts it into chyme. How the soda, the other ingredient in common salt, is disposed of, is not so clear. But as the blood, the bile, and indeed almost all the liquid secretions of the body, except the urine, contain free soda, we can have little doubt about the way that it is finally disposed of.*

"Thus the first step of digestion appears to be the *decomposition of common salt by the nerves of the stomach, and the setting muriatic acid free in a dilute state.* This dilute acid acts as a solvent to the food, and converts it into chyme. The food, thus converted into chyme, is thrown into the duodenum. It would appear that after the solution of the food is once effected, the presence of muriatic acid is no longer useful for the subsequent steps of the digestive process. For in the duodenum, the acid is neutralized by the agency of the bile, which contains a little uncombined soda, but is chiefly characterized by the *picromel,* which has the property of forming an insoluble compound with the muriatic acid, and of neutralizing it. The great use of the bile seems to be to neutralize the muriatic acid, which is so essential in converting the food into chyme."

Dr. Thomson thinks it would be easy to point out "the many important practical results which naturally flow from this theory, and the improvements in the mode of treating dyspeptic and bilious diseases." Now, as the digestion is made to depend on "the decomposition of common salt, by the nerves of the stomach, and the setting muriatic acid free in a dilute state," we conceive that, when the nerves are unable to effect this decomposition, all we have to do is, to introduce into the stomach muriatic acid, in a dilute state—that is to say, in draughts, *tertia quaque hora,* and indigestion will be put to flight! It is with the view of contributing to our stock of knowledge—some people will say, of speculation—and ameliorating some of the greatest evils to which humanity is subject, that we thus endeavour to give the greatest possible extension to the brilliant theory of our Northern confrere.

---

